# Serine ADP-Ribosylation Depends on HPF1

**DOI:** 10.1016/j.molcel.2017.01.003

**Published:** 2017-03-02

**Authors:** Juan José Bonfiglio, Pietro Fontana, Qi Zhang, Thomas Colby, Ian Gibbs-Seymour, Ilian Atanassov, Edward Bartlett, Roko Zaja, Ivan Ahel, Ivan Matic

**Affiliations:** 1 Max Planck Institute for Biology of Ageing, Joseph-Stelzmann-Strasse 9b, Cologne 50931, Germany; 2 Sir William Dunn School of Pathology, University of Oxford, South Parks Road, Oxford OX1 3RE, UK

**Keywords:** ADP-ribosylation, HPF1, PARP-1, PARP-2, histones, proteomics, serine ADP-ribosylation, DNA damage, genome stability

## Abstract

ADP-ribosylation (ADPr) regulates important patho-physiological processes through its attachment to different amino acids in proteins. Recently, by precision mapping on all possible amino acid residues, we identified histone serine ADPr marks in the DNA damage response. However, the biochemical basis underlying this serine modification remained unknown. Here we report that serine ADPr is strictly dependent on histone PARylation factor 1 (HPF1), a recently identified regulator of PARP-1. Quantitative proteomics revealed that serine ADPr does not occur in cells lacking HPF1. Moreover, adding HPF1 to in vitro PARP-1/PARP-2 reactions is necessary and sufficient for serine-specific ADPr of histones and PARP-1 itself. Three endogenous serine ADPr sites are located on the PARP-1 automodification domain. Further identification of serine ADPr on HMG proteins and hundreds of other targets indicates that serine ADPr is a widespread modification. We propose that O-linked protein ADPr is the key signal in PARP-1/PARP-2-dependent processes that govern genome stability.

## Highlights

• Histone serine ADPr depends on HPF1
• HPF1 promotes PARP-1 and PARP-2 serine specificity
• PARP-1 automodifies its serines
• Serine ADPr is a widespread modification

## Graphical Abstract

**Figure:**
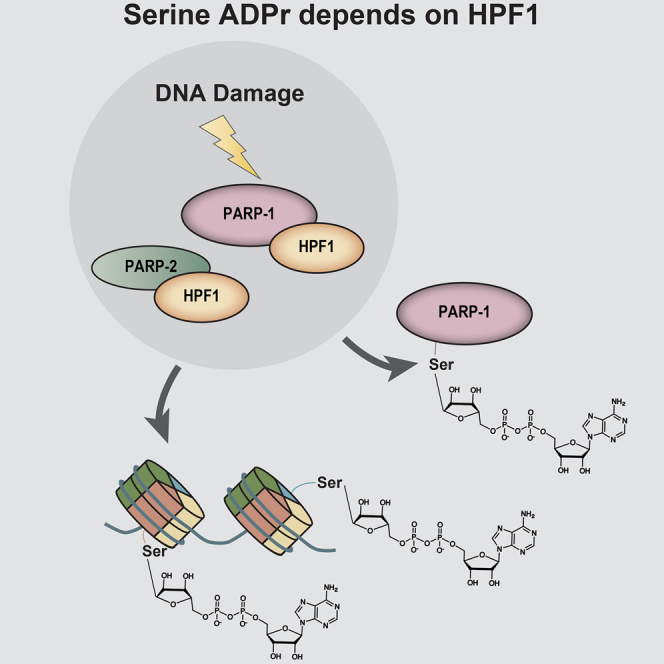

## Teaser

Bonfiglio et al. show that serine residues can be ADP-ribosylated by PARP-1 and PARP-2. This serine specificity is conferred by HPF1, a protein that interacts with PARP-1/2. Serine ADP-ribosylation represents a widespread protein modification that targets hundreds of different substrates, including PARP-1 and high-mobility group proteins.

## Introduction

Many important biological processes are regulated by reversible post-translational modifications (PTMs). One of these, protein ADP-ribosylation (ADPr), is formed by adding adenosine diphosphate ribose molecules to target proteins, modulating their function at different levels, such as subcellular localization, stability, and activity. ADPr regulates many key cellular processes, including maintenance of genomic stability, cell differentiation and proliferation, cytoplasmic stress responses, and microbial virulence (Bock and Chang, 2016, Kraus and Hottiger, 2013, Luo and Kraus, 2012, Rack et al., 2016).

One of the major protein families that catalyze protein ADPr in eukaryotes is poly(ADP-ribose) polymerases (PARPs), alternatively called ADP-ribosyl transferases (ARTDs). PARPs are best understood for their roles in DNA damage response and the regulation of chromatin structure and transcription (Gibson and Kraus, 2012, Martin-Hernandez et al., 2016). The most studied members of this family, PARP-1 and PARP-2 (Langelier and Pascal, 2013), play a critical role during these processes by modifying and recruiting many important chromatin factors, such as histones, p53, topoisomerases, and DNA repair proteins (Caldecott, 2014, Tallis et al., 2014). PARPs modify proteins at specific sites. The general consensus is that acidic residues are the most common targets, but other modified residues, such as lysines, cysteines, and serines, have also been documented (Leidecker et al., 2016, Martello et al., 2016, Messner et al., 2010, Sharifi et al., 2013, Vyas et al., 2014).

Recently, we identified histone PARylation factor 1 (HPF1; also known as C4orf27) as a PARP-1-regulating factor involved in cellular response to DNA damage (Gibbs-Seymour et al., 2016). We have demonstrated that, in the absence of HPF1, PARP-1 is unable to efficiently ADP-ribosylate its main substrates, histones, although the nature of the sites of this HPF1-dependent ADPr remained unresolved.

The analysis of PTMs and their specific target sites has greatly benefited from mass spectrometry (MS)-based proteomics. We recently established a mass spectrometric approach for the unbiased identification of ADPr sites (Leidecker et al., 2016), which provides the methodological platform to investigate HPF1-dependent ADPr. This work identified serine ADPr (S-ADPr) of histones, which is strongly regulated during genotoxic stress, raising the possibility that other proteins involved in DNA damage response, such as PARP-1 itself, could also be modified at serine residues. Here we report that S-ADPr is a widespread modification and that HPF1 acts as a specificity factor for PARP-1 and PARP-2, directing this modification.

## Results

### Histone Serine ADPr Is Dependent on HPF1

**Figure 1:**
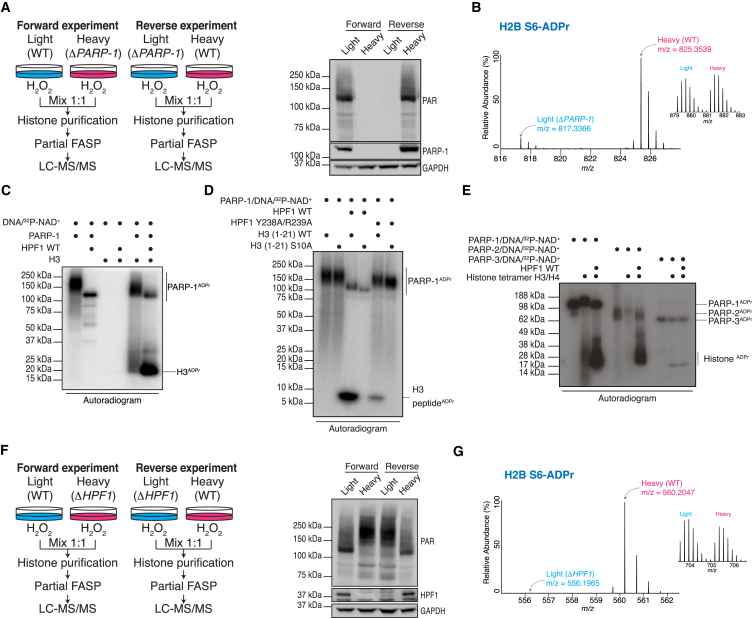
Histone Serine ADPr Is Dependent on HPF1 (A) SILAC strategy to quantify core histone ADPr marks after DNA damage in WT and Δ*PARP*-*1* U2OS cells (left) and a representative western blot of total protein poly-ADP-ribosylation prior to mixing light and heavy lysates from each SILAC experiment (right). Anti-GAPDH was used as a loading control. (B) MS1 of a PARP-1-sensitive modified H2B peptide. The heavy peptide was derived from WT cells, and the light peptide was derived from Δ*PARP*-*1* cells (both stimulated with H_2_O_2_). The inset (right) shows an ∼1:1 ratio (heavy/light) of a non-ADP-ribosylated peptide from the same experiment. (C) Autoradiogram shows ADP-ribosylation of recombinant H3 by PARP-1 in the presence of HPF1. (D) Autoradiogram shows ADP-ribosylation of two synthetic peptide variants corresponding to amino acids 1–21 of human H3. (E) Autoradiogram shows histone tetramer ADP-ribosylation by PARP-1, PARP-2, or PARP-3 in the presence of HPF1. (F) SILAC strategy to quantify core histone ADPr marks upon DNA damage in WT and Δ*HPF1* U2OS cells (left) and a representative western blot of total protein poly-ADP-ribosylation levels prior to mixing light and heavy lysates from each SILAC experiment (right). Anti-GAPDH was used as a loading control. (G) MS1 of an HPF1-sensitive H2B-modified peptide. The heavy peptide was derived from the WT cells, and the light peptide (very low intensity) was derived from Δ*HPF1* cells (both were stimulated with H_2_O_2_). The inset (right) shows an ∼1:1 ratio (heavy/light) of a non-ADP-ribosylated peptide from the same experiment. See also Figure S1.

Recently, we reported the unexpected discovery of ADPr on specific histone serine residues. Modification of these sites is dramatically increased during DNA damage and is blocked by the PARP inhibitor olaparib (Leidecker et al., 2016). Given that PARP-1 is the primary enzyme responsible for ADPr during DNA damage and also the main target of olaparib (Feng et al., 2015, Krishnakumar and Kraus, 2010), we reasoned that PARP-1 could catalyze S-ADPr on histones. To test this hypothesis, we performed a stable isotope labeling by amino acids in cell culture (SILAC; Ong et al., 2002) experiment in cultured human cells (Figure 1A), in combination with a histone purification protocol (Rodriguez-Collazo et al., 2009) and our partial filter-aided sample preparation (partial FASP) digestion to increase the sequence coverage of histones (Leidecker et al., 2016). Strikingly, all detected histone S-ADPr sites were radically decreased in Δ*PARP*-*1* cells compared to wild-type cells (Figures 1B and S1A; Table S1), suggesting that PARP-1 is the major enzyme that ADP-ribosylates histones on serines.

To characterize S-ADPr biochemically, we tried to reconstitute histone modification in vitro using purified components. Intriguingly, we were not able to reproduce S-ADPr in vitro by combining recombinant histones, NAD^+^, activating DNA, and recombinant PARP-1. Under these conditions, histones are comparatively poor ADPr substrates. Since we have previously shown that HPF1 affects the specificity of the PARP-1 reaction toward histones (Gibbs-Seymour et al., 2016) (Figures 1C and S1B), we performed an in vitro reaction of PARP-1/HPF1 with histone H3 as a substrate. The products were analyzed by mass spectrometry with electron-transfer dissociation (ETD) fragmentation. Significantly, we found that H3 was modified on the same serine sites previously identified in cells (Leidecker et al., 2016), namely S10 and S28 (Table S2). Similarly, using histone H1 as a substrate, we observed three previously reported endogenous S-ADPr sites (Table S2). To corroborate HPF1-induced S-ADPr in a more physiological context, we modified human recombinant mononucleosomes in the presence of HPF1. We identified three serine sites on the nucleosome, all of which were found both in cells and on recombinant histones. These experiments show that HPF1 is necessary and sufficient for PARP-1 to ADP-ribosylate histones on serine residues in vitro (Table S2).

S-ADPr of core histones only occurs in the unstructured N-terminal histone tails, as do most canonical histone marks. Since synthetic peptides are commonly used to explore the dynamics of histone marks, especially on the N-terminal tails, we used synthetic H3 peptides to provide an independent confirmation of histone S-ADPr. Peptides corresponding to amino acids 1–21 of human H3 were synthesized as wild-type or an S10A mutant variant, in which the modified residue was replaced by alanine. As observed with full-length histones, no ^32^P radioactivity was incorporated when these peptides were modified by PARP-1 alone. The addition of HPF1 to the reaction induced efficient modification of the H3 wild-type (WT) peptide, while this effect was dramatically reduced for the PARP-1-binding-deficient HPF1 mutants (Y238A/R239A point mutant and the Δ3 mutant with a deleted C terminus) (Figures 1D and S1D) (Gibbs-Seymour et al., 2016). No modification was detected when the S10A mutant peptide was the substrate (Figure 1D), confirming our mass spectrometric identification of S10 as the modified residue (Figure S1C).

Given that HPF1 also interacts with PARP-2 in vivo (Gibbs-Seymour et al., 2016), we reasoned that PARP-2 could have the same effect as PARP-1 when combined with HPF1. Indeed, we observed that PARP-2 is also able to ADP-ribosylate histones in vitro. The effect could be observed for core histones as well as histone H1 and the H3 peptide (Figures 1E, S1B, S1F, and S1G). In contrast, we could not observe the same effect for PARP3 (Figures 1E, S1E, and S1F), another DNA repair PARP that has not been shown to interact with HPF1 (Gibbs-Seymour et al., 2016) and whose catalytic domain is diverged from PARP1/2. Importantly, MS analyses confirmed S-ADPr of histones in the presence of PARP-2, but not for PARP-3 (Table S2).

Next, to investigate the HPF1 dependence of histone S-ADPr in a cellular context, we performed a second SILAC experiment using HPF1-null cells (Figure 1F). Compared to the WT cells, histone S-ADPr was abolished in Δ*HPF1* cells (Figures 1G and S1H; Table S1). For the vast majority of ADP-ribosylated peptides identified in the control cells, no signal was detected in the Δ*HPF1* background, making the calculation of SILAC ratios impossible. Only a few very abundant peptides bearing ADPr on H2BS6 yielded ratios corresponding to an ∼200-fold decrease in ADPr in cells lacking HPF1 (Figure 1G). Collectively, these results confirm our in vitro data and demonstrate that histone S-ADPr is dependent on HPF1.

### HPF1 Changes PARP-1 Amino Acid Specificity toward Serine

The primary targets of PARP-1 ADPr activity are histones and PARP-1 itself (Adamietz, 1987). As shown in Figure 1C, the presence of HPF1 changes the ADPr pattern not only of histones but also of PARP-1. Until recently, PARP-1 ADPr was associated with modification on Asp and Glu residues (Chapman et al., 2013, Sharifi et al., 2013). More recent reports have indicated that Arg and Lys residues are modified residues as well (Daniels et al., 2014, Martello et al., 2016, Messner et al., 2010, Rosenthal et al., 2015), but S-ADPr on PARP-1 had never been reported. Given that HPF1 induces histone S-ADPr, we hypothesized that HPF1 could also have an influence on the amino acid specificity of PARP-1 automodification.

**Figure 2:**
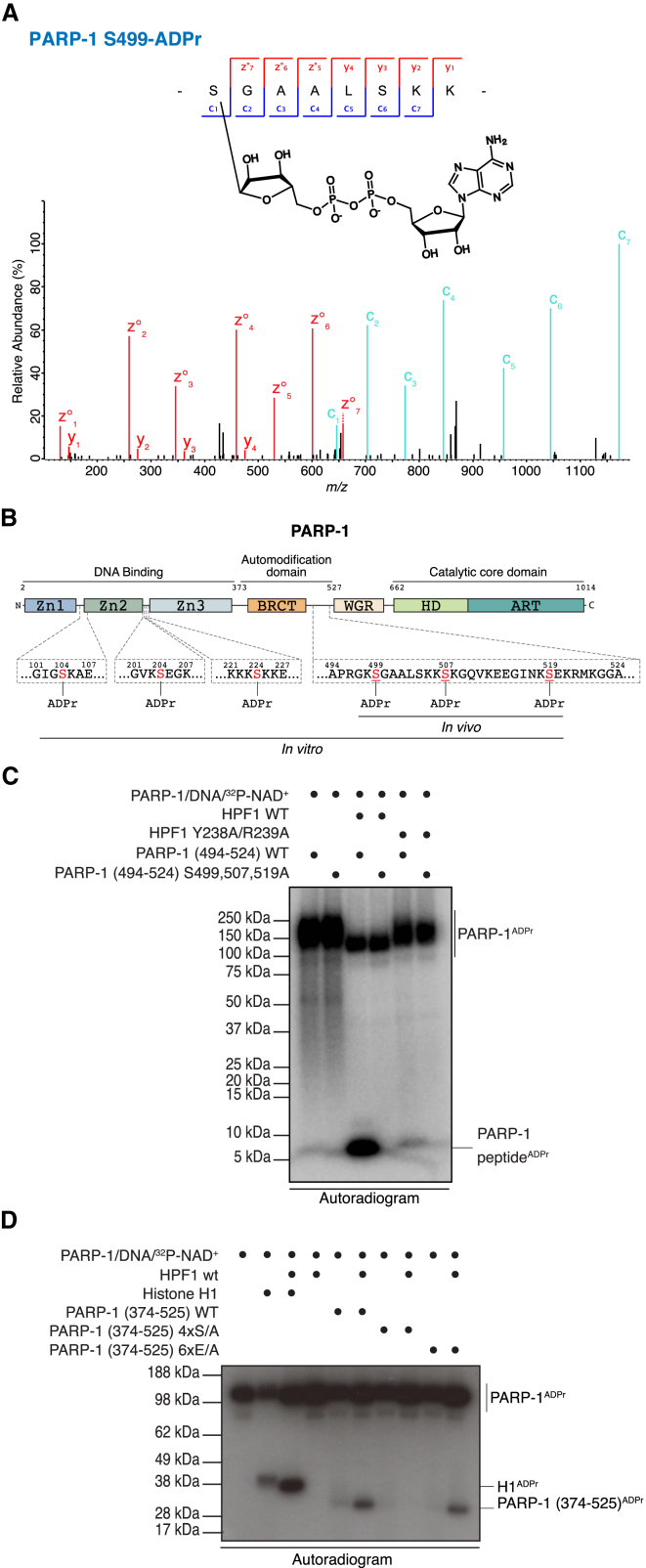
HPF1 Changes PARP-1 Amino Acid Specificity toward Serine (A) High-resolution ETD fragmentation spectrum of a PARP-1 peptide modified by ADP-ribose on serine 499. The chemical structure of ADP-ribose is depicted. (B) Schematic representation of PARP-1 with the six novel serine ADPr sites. The three sites in the unstructured part of the automodificaton region were confirmed in vivo (underlined serines). Zn I/II/III, zinc-finger domains; BRCT, breast cancer suppressor protein-1 domain; WGR, WGR domain; HD, α-helical subdomain; ART, ADP-ribosyl transferase subdomain. (C) Analysis of the ADP-ribosylation of two different synthetic peptides corresponding to amino acids 494–524 of human PARP-1 is shown. (D) Autoradiogram of the ADP-ribosylation of three different variants of the PARP-1 automodification domain (374–525). In vitro ADP-ribosylation of recombinant H1 served as a positive control. See also Figure S2.

To test this, we analyzed the products of in vitro PARP-1 automodification reactions with and without HPF1. To be able to detect poly-ADPr as well as mono-ADPr, we also treated half of each PARP-1 in vitro sample with a Nudix hydrolase (Daniels et al., 2015b, Palazzo et al., 2015). As expected, we did not detect S-ADPr on PARP-1 in the absence of HPF1, but we were still able to confirm previously characterized PARP-1 automodification on acidic residues following the conversion of poly-ADP-ribose to ribose phosphate with Nudix (Figure S2A) (Daniels et al., 2015a). In contrast, we detected S-ADPr on PARP-1 after the addition of HPF1 to the in vitro reaction both with and without subsequent Nudix treatment. Specifically, we identified six S-ADPr sites on PARP-1, three of which lie in the unstructured part of the automodificaton region adjacent to the breast cancer suppressor protein-1 (BRCT) domain (Figures 2A, 2B, and S2B–S2F). Importantly, we confirmed the presence of these three sites (S499, S507, and S519) in vivo by analyzing endogenous PARP-1 purified from cells (Figure 2B). Furthermore, we performed a SILAC experiment using HPF1-null cells to investigate the HPF1 dependence of PARP-1 S-ADPr in a cellular context (Figure S2G). As with histones, PARP-1 S-ADPr was abolished in Δ*HPF1* cells compared to the WT cells (Figure S2H), demonstrating that PARP-1 auto-ADPr on serine is also dependent on HPF1.

Next, we investigated the importance of these sites for HPF1-dependent auto-ADPr of PARP-1 by mutating the serine residues into alanines. Since all detected in vivo sites are located in an unstructured loop of PARP-1 (Langelier et al., 2012), we synthesized peptides corresponding to amino acids 494–524 of human PARP-1, encompassing all three sites. ETD analysis of the native synthetic peptide subjected to in vitro ADPr reactions in the presence of HPF1 confirmed ADPr on S499, S507, and S519 (Table S2). Crucially, HPF1-dependent ADPr was abolished on the peptide when all three serine residues were changed to alanine, as no ^32^P signal was detected (Figure 2C).

To analyze PARP-1 automodification sites in the wider context, we expressed and purified the entire PARP-1 automodification domain (residues 374–525) along with mutants in which either four serines or six glutamate residues were mutated into alanines (4S/A and 6E/A). In vitro reactions on these proteins showed that HPF1-dependent ADPr is dramatically reduced only in the serine mutant (Figure 2D). Taken together, these results suggest that serines in the C-terminal part of the automodification domain are the major sites of PARP-1 automodification in the presence of HPF1.

### Additional Targets of Serine ADPr

Many other proteins are targeted for ADPr (Gibson et al., 2016, Jungmichel et al., 2013, Martello et al., 2016, Zhang et al., 2013), so we set out to identify additional substrates of S-ADPr with two different approaches.

**Figure 3:**
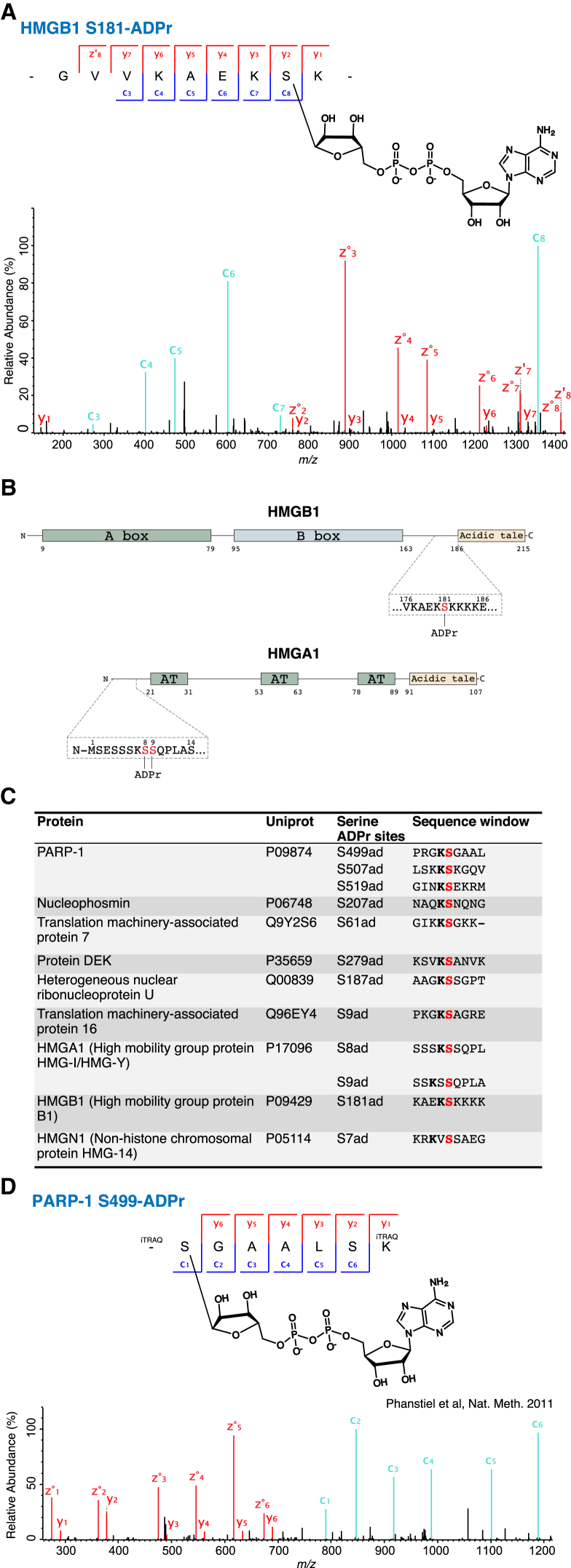
Additional Targets of Serine ADPr (A) High-resolution ETD fragmentation spectrum of an HMGB1 peptide with ADP-ribose on serine 181 is shown. (B) Schematic representations of HMGB1 (upper) and HMGA1 (lower). The three novel serine ADPr sites were identified in vivo. A box and B box, positively charged homologous DNA-binding structures; acidic tail, negatively charged region composed of 30 glutamic and aspartic acids, exclusively; AT, AT-hook with the Arg-Gly-Arg-Pro (RGRP) core motif. (C) Serine ADPr sites identified in histone-depleted fractions. ADPr sites were identified using ETD mass spectrometry. Modified serines are in red. (D) High-resolution ETD fragmentation spectrum of a PARP-1 peptide with ADP-ribose on serine 499, obtained by reprocessing a published dataset (Phanstiel et al., 2011). The chemical structure of ADP-ribose is depicted. See also Figure S3.

First, we employed our published histone purification protocol (Leidecker et al., 2016) to search for ADPr in fractions depleted of histones. This method prevents artifactual enzymatic ADPr and non-enzymatic ADPr by lysing cells under denaturing conditions. We examined the fraction depleted in histones, since their ADPr sites have already been characterized (Leidecker et al., 2016) and could only interfere with the identification of ADPr sites on other less abundant proteins. Proteins were digested by partial FASP to provide more ETD-compatible peptides (Leidecker et al., 2016). High-resolution ETD analysis confirmed ADPr on S499, S507, and S519 in PARP-1, and it revealed five targets of S-ADPr as follows: HMGA1 (S8 and S9), HMGB1 (S181), HMGN1 (S7), NPM1 (S207), and TMA7 (S61) (Figures 3A–3C, S3A, and S3B). As this approach does not specifically enrich for ADPr, it only detects abundant ADPr sites. The identified substrates are thus likely to be among the main targets of ADPr. To increase the sensitivity of our analysis and identify more targets, we combined this fractionation protocol with phosphopeptide enrichment adapted to ADPr (Chapman et al., 2013, Daniels et al., 2014). This yielded the following three additional S-ADPr substrates: DEK (S279), HNRNPU (S187), and TMA16 (S9) (Figures 3C, S3C, and S3D).

Second, we reasoned that reprocessing published, high-quality proteomic datasets could reveal previously overlooked S-ADPr sites from unidentified spectra (Matic et al., 2012). As illustrated by a recent study (Griss et al., 2016), computational strategies for mining published proteomic data have particularly benefited the discovery of unexpected protein modifications. To confirm our findings and investigate the scope of S-ADPr further, we reprocessed a high-quality phosphoproteomic study of human stem cells from the Coon group (Phanstiel et al., 2011). Importantly, a high-quality ETD spectrum unidentified in the original study provided independent confirmation of our identification of PARP-1 ADPr on S499 (Figure 3D). Our reanalysis also confirmed two histone ADPr marks (H2BS6 and H3S28) and S-ADPr of HMGA1, although the corresponding higher-energy collision-induced dissociation (HCD) spectrum did not differentiate between modifications on S8 and S9 (both of which were identified as ADPr sites by our in vivo ETD analyses described above).

### Serine ADPr Is a Widespread PTM

As shown above, S-ADPr is not restricted to histones but also targets PARP-1 and at least eight additional substrates, including three chromosomal high-mobility group proteins. Thus, we hypothesized that S-ADPr might be a widespread PTM. Recent large-scale proteomic studies have identified thousands of additional proteins as targets of ADPr in the DNA damage response, but the site specificity of these analyses has been, to a large extent, limited to Asp, Glu, Lys, and Arg (Daniels et al., 2015a, Gibson et al., 2016, Jungmichel et al., 2013, Martello et al., 2016, Vivelo and Leung, 2015, Zhang et al., 2013). This raises the question of whether some known ADP-ribosylated proteins may be modified on serine residues.

To test this hypothesis, we applied our reanalysis approach to a recent site-specific ADPr proteomic study based on the enrichment of modified peptides with a macrodomain ADPr-binding module (Martello et al., 2016). Although the enrichment strategy used in this study was not limited to modifications on particular amino acids (Gibson et al., 2016, Zhang et al., 2013), the data search parameters restricted ADPr to Asp, Glu, Lys, and Arg, but not other amino acids that have been suggested as targets of ADPr (Daniels et al., 2015a). Motivated by our unexpected identification of cysteine ADPr (Vyas et al., 2014), we broadened our computational analyses to consider ADPr on all reactive amino acids. This approach made our discovery of S-ADPr possible (Leidecker et al., 2016). Since the search parameters employed by Martello et al. (2016) preclude the discovery of ADPr on serine residues, we expected that S-ADPr could be hidden in the HCD raw files that they have made publicly available.

While HCD consistently produces diagnostic ions demonstrating the presence of ADPr (Hengel and Goodlett, 2012), it is poor for localization of this modification, especially the extremely fragile serine linkage (Leidecker et al., 2016). Standard-quality HCD spectra of ADP-ribosylated peptides contain few ions localizing the modification; but, given that Martello et al. (2016) employed a powerful enrichment approach and performed data acquisition at higher resolution and sensitivity, we reasoned that reprocessing these particular HCD files could allow the identification of some S-ADPr sites, though with lower localization confidence than that provided by ETD analyses (Hengel and Goodlett, 2012). To identify S-ADPr sites in these data, we manually inspected the matched spectra for information pinpointing ADPr on serine residues (see the STAR Methods). When ADPr on serine was allowed, the dataset generated by Martello et al. (2016) yielded over 250 localized S-ADPr sites (Table S3).

**Figure 4:**
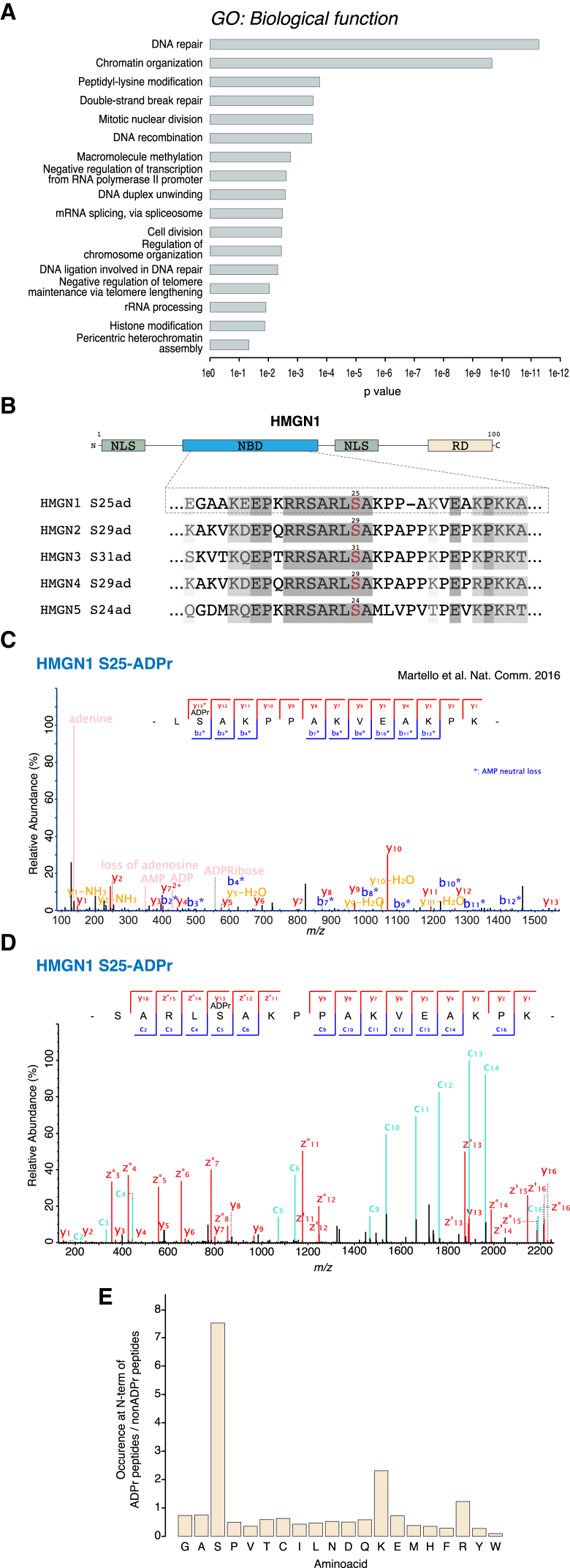
Serine ADPr Is a Widespread PTM (A) Gene ontology analysis of the serine ADPr proteins obtained by reprocessing a published dataset (Martello et al., 2016). Biological processes enriched in the serine ADPr proteins are shown. (B) Schematic representation of HMGN1 (upper panel) and sequence alignment of the highly conserved N-terminal region of the HMGN family (lower panel). Note that the serine ADPr site (red) is conserved. NLS, nuclear localization signal; NBD, nucleosomal binding domain; RD, regulatory domain. (C) HCD fragmentation spectrum of an HMGN1 peptide modified by ADP-ribose, obtained by reprocessing a published dataset (Martello et al., 2016). This HCD spectrum contains sufficient localization information to assign serine 25 as the modified residue. ^∗^AMP neutral loss. (D) High-resolution ETD fragmentation spectrum of an HMGN1 peptide ADP-ribosylated on serine 25 in vitro in the presence of PARP-1 and HPF1 is shown. (E) Bar plot shows the occurrence of the different amino acids at N termini of ADPr peptides relative to non-ADPr peptides, obtained by reprocessing a published dataset (Martello et al., 2016). See also Figure S4.

Gene ontology analysis of all of the above data combined revealed that S-ADPr is strongly enriched in proteins involved in processes maintaining genome stability, such as DNA repair and replication, transcription, mRNA splicing, and the regulation of chromatin structure and mitosis (Figure 4A). This analysis also suggested a number of other protein functions commonly regulated by ADPr (Table S3) (Daniels et al., 2015a). These include chromatin remodelers, histone and DNA methylation systems, proteins central to a variety of DNA repair pathways (DNA ligase, DNA-PKScs, BLM, XPC, MSH6, Polbeta, FANCI, and Rad54B), high-mobility group (HMG) and heterogeneous ribonucleoprotein particle (HNRNP) proteins, and many zinc-finger-containing factors.

S-ADPr of high-mobility group proteins appears to be characteristic, since we found that all three different families (HMGA, HMGB, and HMGN) are modified by ADPr on serine residues (Figure 3C). Our reanalysis of the dataset generated by Martello et al. (2016) confirmed S-ADPr sites on HMGA1 and HMGN1 identified by our analyses of histone-depleted fractions. Strikingly, all five members of the high-mobility group nucleosome-binding (HMGN) family were serine ADP-ribosylated in this dataset, and on all of them the modified serine was located in a highly conserved N-terminal region (Figures 4B and 4C; Table S3). This further strengthens our suggestion that chromatin factors of the high-mobility group are among the main substrates of S-ADPr in vivo. Using purified HMGA1, HMGB1, HMGN1, HMGN2, and HMGN4, we could reproduce the in vivo sites (HMGA1 [S8 and S9], HMGB1 [S181], HMGN1 [S25], HMGN2 [S29], and HMGN4 [S29]) in vitro in the presence of PARP-1 and HPF1 (Figures 4D and S4).

In addition to pinpointing ADPr on over 250 serine residues in the HCD dataset generated by Martello et al. (2016), we also re-analyzed the data disregarding the limited localization information that HCD provides (see the STAR Methods for details). We observed that a disproportionate number of ADP-ribosylated peptides identified by this unlocalized search contain serine and, more specifically, a serine preceded by a basic residue in the protein sequence. This conforms to the primitive consensus motif we proposed for histone sites (Leidecker et al., 2016) and have further refined with the in vivo sites observed in this study (Figures 2B, 3B, and 3C). Since basic residues are trypsin cleavage sites, the modified serine is likely to be the first residue of a fully cleaved tryptic peptide. An analysis of the frequency of the first amino acids of the identified peptides (see the STAR Methods for details) revealed a gross over-representation of serine in the first position of modified peptides compared to non-modified ones (Figure 4E; Table S4). This indicates that many of the ADP-ribosylated peptides enriched by Martello et al. (2016) are likely modified on serine residues matching the proposed motif, and it shows that S-ADPr may be much more widespread than previously thought and well beyond the almost 300 sites identified above.

## Discussion

The covalent attachment of diverse PTMs to specific amino acid residues within proteins is the molecular basis for increasing the functional diversity of a proteome. The best-known PTMs, such as acetylation and phosphorylation, involve the attachment of small chemical groups. Recent technical advances, however, have allowed us to broaden our attention to increasingly complex PTMs. A prominent feature of larger PTMs is the functional versatility created by their structural complexity. For ADPr, versatility also arises from its broad amino acid specificity.

In some cases, this broad amino acid specificity appears to be a property of a family of enzymes or even a single enzyme, as in the case of PARP-1, raising the question of how an enzyme can create diverse conjugation chemistries (Daniels et al., 2015a) and also catalyze highly specific reactions. We recently expanded the repertoire of ADP-ribosylated amino acids with the discovery of S-ADPr (Leidecker et al., 2016). Our current study reveals the biochemical basis for S-ADPr, identifying HPF1 as the factor conferring serine specificity on both PARP-1 and PARP-2. Since PARP-1 is more abundant than HPF1 in cells (Gibbs-Seymour et al., 2016), it is likely that PARP-1 forms other protein complexes as well, in which other partners could steer PARP-1 and other PARPs to modify targets on different sites. Thus, our work provides a possible template for future searches for factors responsible for other types of ADP-ribose-protein attachments.

Curiously, although the attachment of ADP-ribose to serine is chemically identical to the linkage between ADP-ribose monomers in poly-ADP-ribose (both are acetal bonds), ADP-ribose polymerization is clearly independent of HPF1. This suggests that the formation of the acetal bond on proteins ADP-ribosylated by PARP-1 is mechanistically different from the creation of the acetal linkages between molecules of ADP-ribose. DNA damage destabilizes the catalytic domain of PARP-1, which leads to poly-ADPr of PARP-1 (Langelier et al., 2012). Similar structural studies are needed in the future to provide the structural basis for the HPF1-induced change of amino acid specificity of PARP-1.

Our work establishes S-ADPr as a widespread modification in PARP signaling by identifying hundreds of protein targets for this type of ADPr. Specifically, our data suggest that serine is a much more common ADPr target than previously considered and that this signal is particularly utilized by processes important for genome stability. The identified serine sites on the primary substrates, histones, and PARP-1 itself have been shown to be dependent on HPF1, suggesting that HPF1 has a profound effect on PARP activity. Moreover, this specificity seems to extend beyond these primary targets to other substrates, including HMG proteins. HMG proteins have important roles in chromatin regulation, DNA repair, and cancer progression (Reeves, 2015). Strikingly, the ADPr site shared by all HMGNs is at the interface between HMGNs and nucleosome core proteins (Prymakowska-Bosak et al., 2001).

We have reason to believe that many additional O-linked ADPr sites (on serine and possibly threonine and tyrosine residues) await discovery, since standard proteomic approaches for the identification of ADPr sites have generally not combined three key elements. First, an essential component of such a strategy is computational analysis that permits identification of O-ADPr sites. Second, while our data analysis can sometimes yield localizations from sensitive HCD data, the approach works best with high-resolution ETD fragmentation, which preserves ADPr (Hengel and Goodlett, 2012). Third, since around half of the sites we have identified unambiguously in vivo by ETD on histones, PARP-1 and four other substrates fall in Lys- and Arg-rich regions, the detection of these sites is hampered by standard digestion protocols. In addition, ETD is inefficient with conventional tryptic peptides, while partial FASP generates longer peptides ideally suited for this fragmentation mode (Leidecker et al., 2016). Thus, we propose that thousands of O-linked ADPr sites will be identified, once our partial FASP protocol and data analysis strategy are combined with high-resolution ETD and a powerful biochemical enrichment of ADP-ribosylated peptides.

In summary, we provide the molecular mechanism for the recently discovered S-ADPr (Leidecker et al., 2016). We identify HPF1 as the S-ADPr-inducing factor, and we show that S-ADPr is a widespread PTM that targets hundreds of proteins, including PARP-1 itself. We speculate that O-linked ADPr on serine (and possibly threonine and tyrosine) is the major type of ADPr in the regulation of DNA damage response and the maintenance of genome stability.

## STAR★Methods

### Key Resources Table

**Table:** 

REAGENT or RESOURCE	SOURCE	IDENTIFIER
**Antibodies**		

Amersham ECL Rabbit IgG, HRP-linked whole Ab	GE Healthcare	Cat# NA934
Amersham ECL Mouse IgG, HRP-linked whole Ab	GE Healthcare	Cat# NA931
Anti-PAR Polyclonal Antibody (rabbit)	Trevigen	Cat# 4336-BPC-100
Anti-PARP antibody	Abcam	Cat# ab6079
Anti-HPF1 polyclonal antibodies	Gibbs-Seymour et al., 2016	N/A
Anti-GAPDH Mouse mAb (6C5)	Merck Millipore	Cat# CB1001

**Chemicals, Peptides, and Recombinant Proteins**		

Unlabelled L-lysine (Light)	Sigma-Aldrich	Cat# L8662
Isotopically labeled L-lysine (^13^C6,^15^N2)	Sigma-Aldrich	Cat# 608041
Sulfopropyl (SP)-Sepharose resin	Sigma-Aldrich	Cat# S1799
PHOS-Select Iron Affinity Gel	Sigma-Aldrich	Cat# P9740
cOmplete Protease Inhibitor Cocktail	Sigma-Aldrich	Cat# 000000004693116001
Amersham ECL Select Western Blotting Detection Reagent	GE Healthcare	Cat# RPN2235
Trypsin Gold, Mass Spectrometry Grade	Promega	Cat# V5280
Nudix 16 hydrolase	Palazzo et al., 2015	N/A
Recombinant PARP-1 high specific activity	Trevigen	Cat# 4668
Recombinant human histone H3	NEB	Cat# M2503S
Recombinant human histone H1.0	NEB	Cat# M2501S
Recombinant human histone H1.2 (Human)	Novus Biologicals	Cat# H00003006-P01
Recombinant human mononucleosomes	EpiCypher	Cat# 16-0009
Recombinant human HMGN1	Abnova	Cat# H00003150-P01
Recombinant human HMGN2	Abnova	Cat# H00003151-P01
Recombinant human HMGN4	Abnova	Cat# H00010473-P01
Recombinant human HMGB1	RayBiotech	Cat# 228-10767-2
Histone tetramers, octamers and nucleosomes	Mehrotra et al., 2011	N/A
Recombinant human PARP-1	Langelier et al., 2011	N/A
Recombinant human PARP-2	Langelier et al., 2014	N/A
Recombinant human HMGA1	This study	N/A
Recombinant human PARP-3	This study	N/A
PARP-1 (374-525) fragment wild type, 4xS-A and 6xE-A mutants	This study	N/A
HPF1 WT, Y238A/R239A and HPF1 Δ3	Gibbs-Seymour et al., 2016	N/A
Activated DNA	Trevigen	Cat# 4671-096-06
NAD^+^	Trevigen	Cat# 4684-096-02
^32^P-NAD^+^	Hartmann Analytic	Cat# ARP 0141
Olaparib	Cayman Chemical	Cat# 10621
ADP-HPD, Dihydrate, Ammonium Salt - Calbiochem	Merck Millipore	Cat# 118415
3-ABA - CAS 3544-24-9 - Calbiochem	Merck Millipore	Cat# 165350
H3 (1-21) WT: Ac-ARTKQTARKSTGGKAPRKQLAGGA-Am	This study	N/A
H3 (1-21) S10A: Ac-ARTKQTARKATGGKAPRKQLAGGA-Am	This study	N/A
PARP-1 (494-524) WT: Ac-APRGKSGAALSKKSKGQVKEEGINKSEKRMKGGA-Am	This study	N/A
PARP-1 (494-524) S499,507,519A: Ac- APRGKAGAALSKKAKGQVKEEGINKAEKRMKGGA-Am	This study	N/A

**Critical Commercial Assays**		

Commercial PARP-1 nanotrap	Chromotek	Cat# xta-20

**Deposited Data**		

Unprocessed image files used to prepare the figures in this manuscript and manually validated spectra from the reanalysis of Martello et al., 2016 dataset	This study	http://dx.doi.org/10.17632/pmvv5mdmrm.1
Mass-spectrometry data	This study	ProteomeXchange: PXD005627
Mass spectrometry data: phosphoproteomics study of human stem cells	Phanstiel et al., 2011	Stem Cell–Omics Repository (SCOR; http://scor.chem.wisc.edu/)
Mass spectrometry data: enrichment of modified peptides with a macrodomain ADPr-binding module.	Martello et al., 2016	ProteomeXchange: PXD004245

**Experimental Models: Cell Lines**		

Human: U2OS cells	ATCC	HTB-96
Human: U2OS cells ΔPARP-1	Gibbs-Seymour et al., 2016	N/A
Human: U2OS cells ΔHPF1	Gibbs-Seymour et al., 2016	N/A

**Recombinant DNA**		

PARP-1 and PARP-2 expression constructs	Gift from John Pascal (University Montreal)	N/A
HPF1 WT, Y238A/R239A and HPF1 Δ3	Gibbs-Seymour et al., 2016	N/A
HMGA1	This study	N/A
PARP-3	This study	N/A
PARP-1 (374-525) fragment wild type, 4xS-A and 6xE-A mutants	This study	N/A

**Software and Algorithms**		

MaxQuant proteomics suite of algorithms (version 1.5.3.17)	Cox and Mann, 2008	http://www.maxquant.org
Perseus software	Tyanova et al., 2016	http://www.perseus-framework.org
Morpheus 1.68	Wenger and Coon, 2013	http://cwenger.github.io/Morpheus/
Panther	Mi et al., 2013	http://www.pantherdb.org/panther/ontologies.jsp

**Other**		

10 kDa cut-off Vivacon 500 flat filters	Sartorius	Cat# VN01H03

### Contact for Reagent and Resource Sharing

Further information and requests for resources and reagents should be directed to and will be fulfilled by the Lead Contact, Dr. Ivan Matic (imatic@age.mpg.de).

### Experimental Model

#### Cell Culture and SILAC Labeling

U2OS cells (wild-type and knockout cell lines) were cultured in Dulbecco’s modified Eagle’s medium (DMEM) supplemented with 10% fetal bovine serum, penicillin/streptomycin (100U/ml) at 37°C, 5% CO2. Cells were regularly tested for Mycoplasma by PCR-based detection analysis and discarded if positive.

For SILAC labeling (Ong et al., 2002), U2OS cells (wild-type and knockout cell lines) were grown in medium containing unlabelled L-lysine as the light condition, or isotopically labeled L-lysine (^13^C6,^15^N2) as the heavy condition. Both light and heavy DMEM were supplemented with 10% dialyzed FBS (Thermo Scientific). Cells were cultured for more than 7 generations to achieve complete labeling. Incorporation efficiency (> 99%) was determined by MS.

### Method Details

#### Histone Purification

Histones were purified as previously described (Leidecker et al., 2016). Briefly, after H_2_O_2_ stimulation, cells were washed twice with ice-cold PBS and lysed by rotation in 0.1 M H_2_SO_4_ at 4°C for 2 hr. The lysate was centrifuged at 2200 g at 4°C for 20 min. The pellet with non-soluble proteins and cell debris was discarded. Sulfuric acid-soluble proteins were neutralized with 1M Tris-HCl pH 8.0. NaCl, EDTA and DTT were added to a final concentration of 0.5 M, 2 mM, 1 mM, respectively. For ion exchange chromatography, sulfopropyl (SP)-Sepharose resin was packed into a column and pre-equilibrated with 10 volumes of Binding Buffer (50 mM Tris-HCl pH: 8.0, 0.5 M NaCl, 2 mM EDTA, 1 mM DTT). The neutralized supernatant containing H_2_SO_4_ -soluble proteins was passed through the column. The resin was washed with 10 volumes of Binding Buffer and 30 volumes of Washing Buffer (50 mM Tris-HCl pH 8.0, 0.6 M NaCl, 2 mM EDTA, 1 mM DTT). Proteins were eluted with Elution Buffer (50 mM Tris-HCl pH 8.0, 2 M NaCl, 2 mM EDTA, 1 mM DTT) in 10 fractions. Eluted proteins (mainly core histones) were precipitated overnight in 4% (v/v) PCA at 4°C. The fractions were then centrifuged at 21,000 g at 4°C for 45 min and the resulting pellets were washed with 4% PCA (2 × 1 ml), 0.2% HCl in acetone (2 × 1 ml), acetone (2 × 1 ml).

#### Strategy for Identification of Additional Substrates of Serine ADPr

In order to identify additional substrates of serine ADPr we employed the typically discarded fractions of the above-mentioned purification strategy (depleted of core histones), since ADPr of histones had already been characterized (Leidecker et al., 2016) and could only interfere with identification of ADPr sites on other less abundant proteins. Proteins from histone-depleted fractions were precipitated overnight in 30% (v/v) Trichloroacetic acid (TCA) at 4°C. The fractions were then centrifuged at 21,000 g at 4°C for 45 min and the resulting pellets were washed with 30% TCA (2 × 1 ml), 0.2% HCl in acetone (2 × 1 ml), acetone (2 × 1 ml).

#### Enrichment of ADPr Peptides from Histone-Depleted Fractions with Immobilized Metal Affinity Chromatography (IMAC)

The enrichment was performed as in Daniels et al. (Daniels et al., 2014), with some modifications. Peptides were resuspended in 40% ACN, 0.1% FA (binding buffer) and incubated with 10 μL PHOS-select beads for 1 hr, with end-to-end rotation at 25°C. The beads were then spun down and the supernatant was further incubated with 10 μL of beads. The bead-bound peptides were washed once with binding buffer and transferred to a pre-equilibrated StageTip. There, the beads were washed twice with binding buffer and acidified with 1% FA. Peptides were eluted onto the StageTip with 0.5 M potassium phosphate pH 7, acidified again with 1% FA and washed with 0.1% FA. They were eluted with 40% ACN, 0.1% FA and dried down in the SpeedVac concentrator.

#### SILAC Experiments

For histones analysis, cells were stimulated with 2 mM H_2_O_2_ for 10 min, washed twice with ice-cold PBS and lysed by rotation in 0.1 M H_2_SO_4_ at 4°C for 2 hr. Prior to centrifugation at 4°C, aliquots from the light and heavy lysates were retained for western blot analysis. Supernatants containing sulfuric acid-soluble fractions from light and heavy lysates were mixed 1:1 and histones were purified as described above.

For PARP-1 analysis, cells were stimulated with 2 mM H_2_O_2_ for 10 min, washed twice with ice-cold PBS and lysed by rotation in Lysis buffer (10 mM Tris/Cl pH 7.5; 150 mM NaCl; 0,5 mM EDTA; 0.5% NP-40), supplemented with protease inhibitor cocktail, 1 μM ADP-HPD, 2 μM olaparib and 10 mM 3-aminobenzamide (3-ABA). After a centrifugation step (10 min at 20,000 x g), supernatants from light and heavy lysates were mixed 1:1 and PARP-1 was purified as described below.

Each SILAC experiment was composed of at least two biological replicates.

#### Western Blot Analysis

For western blot analysis, samples were subjected to a standard SDS-PAGE method. Proteins were transferred to PVDF membranes (Merck Millipore). Membranes were then blocked with TBS-T buffer (25 mM Tris-HCl pH 7.5, 150 mM NaCl, 0.05% Tween 20 and 5% non-fat dried milk) and probed overnight with primary antibodies at 4°C, followed by a one hour incubation with peroxidase-conjugated secondary antibodies at room temperature. Blots were developed using ECL Select and signals were captured using a ChemiDoc MP System (Bio-Rad). Dilutions used for the primary antibodies were: Anti-poly-ADP-ribose: diluted at 1:2000, anti-PARP-1: diluted 1:1000, anti-HPF1: diluted 1:500, anti-GAPDH: diluted at 1:2000. Loading controls (GAPDH) were run on the same blot as poly-ADP-ribose blots.

#### Protein Digestion

Proteins were digested using partial FASP as previously described (Leidecker et al., 2016). Briefly, proteins were resuspended in 200 μL of 8 M urea in 0.1 M Tris-HCl pH 8.0, 10 mM tris(2-carboxyethyl)phosphine (TCEP), 20 mM chloroacetamide and transferred to 10 kDa cut-off Vivacon 500 flat filters. Samples were centrifuged at 14,000 g at 20°C for 20 min, followed by three washes with 200 μL of 50 mM ammonium bicarbonate (ABC).

For partial FASP digestion, 1:2000 Trypsin Gold to protein ratio was used for 20 min at 20°C. The digestion was stopped by the addition of formic acid to lower the pH below 3. Peptides were collected by centrifugation at 14,000 g at 4°C for 10 min. Next, 50 μL of 50 mM ABC were added to the filter and peptides were collected by centrifugation at 14,000 g at 4°C. This elution step was repeated.

The retentate containing undigested proteins was further digested in 50 μL 50 mM ABC overnight at 37°C, with 1:50 trypsin to protein ratio. Peptides were collected by centrifugation at 14,000 g at 4°C for 10 min, followed by a further elution with 50 μL 50 mM ABC.

Peptides were then desalted on either C18 cartridges (3M Empore) or using in-house manufactured StageTips (Rappsilber et al., 2003), depending on the peptide amounts. Eluted peptides were dried down in Speedvac concentrator and resuspended in 0.1% FA prior to LC-MS/MS analysis.

#### Variation of the Digestion Method for In Vitro Automodified PARP-1

After in vitro automodification of 2μM PARP-1 in the presence or absence of 2 μM HPF1 WT, 200 μL of 8 M urea in 0.1 M Tris-HCl pH 8.0, 10 mM tris(2-carboxyethyl)phosphine (TCEP), 20 mM chloroacetamide were added to the reaction and samples were transferred to 10 kDa cut-off Vivacon 500 flat filters. Samples were centrifuged at 14,000 g at 20°C for 20 min, followed by three washes with 200 μL of Nudix reaction buffer (50 mM Tris-HCl pH 8.0, 15 mM MgCl_2_, 50 mM NaCl, 0.1 mM TCEP). Protein digestion was performed in 50 μl of Nudix reaction buffer on top of the filter by adding Trypsin Gold in 1:100 to protein ratio for 30 min at 20°C. Peptides were collected by centrifugation at 14,000 g at 4°C for 10 min. Next, 50 μL of Nudix reaction buffer were added to the filter and peptides were collected by centrifugation at 14,000 g at 4°C. This elution step was repeated. Then, two washes with 200 μl of 8M Urea followed by centrifugation at 14,000 g at 4°C were performed to reduce trypsin activity. After centrifugation, three washes with 200 μl of Nudix reaction buffer were performed. Finally, eluted peptides from partial FASP digestion were loaded onto the FASP filter and processed, when indicated, with Nudix 16 hydrolase (see below).

#### Hydrolysis of ADP-Ribosylated Peptides by Nudix16

In order to render poly-ADP-ribosylation sites amenable to MS analysis without the risk of non-enzymatic ADPr due to high levels of free ADP-ribose, PARP-1 peptides were processed, when indicated, with Nudix 16 hydrolase, which converts both ADPr and poly-ADPr to phosphoribose and free AMP, which cannot lead to re-elongation (Daniels et al., 2015b, Palazzo et al., 2015).

Eluted peptides from partial FASP digestion were loaded onto the FASP filter and incubated, when indicated, with 30 mM recombinant Nudix16 (Daniels et al., 2015b, Palazzo et al., 2015) at RT for 1 hr. Peptides were collected by centrifugation at 14,000 g at 4°C for 10 min. Next, 50 μL of Nudix reaction buffer were added to the filter and peptides were collected by centrifugation at 14,000 g at 4°C. This elution step was repeated. Peptides were then desalted on either C18 cartridges (3M Empore) or using in-house manufactured StageTips (Rappsilber et al., 2003), depending on the peptide amounts. Eluted peptides were dried down in Speedvac concentrator and resuspended in 0.1% FA prior to LC-MS/MS analysis.

#### In Vitro ADP-Ribosylation Assays

In vitro ADP-ribosylation assays were performed as previously described (Gibbs-Seymour et al., 2016). For autoradiography analyses, recombinant proteins or synthetic peptides were mixed with PARP-1 and/or HPF1 WT or mutants before the addition of activated DNA, 5 μM NAD^+^ and 63nM (0.05 μCi/μl) ^32^P-NAD^+^ to the reaction, which proceeded for 20 min at room temperature. Olaparib (2μM final concentration) was added at the end of the reactions before subsequent analysis by autoradiography. The molarity of HPF1 proteins used in the reactions were 1-2 μM, PARP-1 or PARP-2 were 0.1 μM and trans substrates were typically used at 2μM. The recombinant H3 and synthetic peptides substrates were 2μg per condition.

For mass spectrometry analyses, recombinant proteins or recombinant human mononucleosome or synthetic peptides were mixed with PARP-1 (Trevigen) or in-house produced recombinant PARP-1 or PARP-2 from *E.coli*, and/or HPF1 WT before the addition of activated DNA and 200 μM NAD^+^ to the reaction, which proceeded for 20 min at room temperature. Olaparib (2μM final concentration) was added at the end of the reactions before subsequent trypsin digestion. The molarity of HPF1 WT used in the reactions were 2 μM, PARP-1 and PARP-2 was 0.1 μM. The mass of substrates that we were interested in analyzing their modification sites ranged from 1 to 50 μg per reaction.

#### PARP-1 Immunoprecipitation

For immunoprecipitation of endogenous PARP-1 we used a commercial PARP-1 nanotrap according to the manufacturer’s instructions. Briefly, after H_2_O_2_ stimulation, cells were washed twice with ice-cold PBS and lysed by rotation in Lysis buffer (10 mM Tris/Cl pH 7.5; 150 mM NaCl; 0,5 mM EDTA; 0.5% NP-40), supplemented with protease inhibitor cocktail, 1 μM ADP-HPD, 2 μM olaparib and 10 mM 3-ABA. After a centrifugation step (10 min at 20,000 x g), the soluble fraction was adjusted with dilution buffer (10 mM Tris/Cl pH 7.5, 150 mM NaCl) supplemented with protease inhibitor cocktail, 1 μM ADP-HPD, 2 μM olaparib and 10 mM 3-ABA. Next, lysate was incubated with the PARP-1 nanotrap for 1 hr in an end-over-end rotor at 4°C. The bead pellet was washed three times in dilution buffer. After the last washing step, PARP-1 nanotrap beads were resuspended in 8 M Urea solution by pipetting up and down, incubated for 5 min at room temperature while shaking at 700 rpm, and centrifuged at 2.500x g for 2 min at RT. Supernatant was transferred to a new tube. This elution step was repeated once.

All immunoprecipitated complexes were digested with the partial FASP method and analyzed by mass spectrometry, as described.

#### LC-MS/MS Analysis

Liquid chromatography for all LC-MS/MS runs was performed on an EASY-nLC 1000 Liquid Chromatography system (Thermo Scientific) coupled to the spectrometers via modified NanoFlex sources (Thermo scientific). Peptides were loaded onto 250-mm x 75-μm PicoFrit (C18 2 μm medium) analytical columns (New Objective) at a maximum pressure of 800 bar. Solutions A and B for the UPLCs were 0.1% formic acid in water and acetonitrile, respectively. Samples were loaded in 0.1% formic acid in water to maximize retention of highly hydrophilic peptides. Gradients varied slightly in length (90 to 150 min) and mixture, and may be extracted from the respective raw files. In general they incorporated a linear gradient from very low or zero %B to 20 or 30% for 65-100 min, followed by a steeper phase and a wash. This length of gradient was maintained despite the relative simplicity of the protein mixture in order to improve the resolution and identification of as many modified peptide forms as possible, including those of low abundance.

#### HCD (Higher-Energy Collision-Induced Dissociation) Acquisitions

Pure HCD datasets were acquired on a Q Exactive Plus HF mass spectrometer (Thermo Scientific). Optimal data acquisition for our low-complexity samples was achieved by increasing the sensitivity of the analysis (Kelstrup et al., 2012). MS1 spectra were acquired in the 300-1800 m/z scan range with a resolution of 120,000. AGC targets were set to 3,000,000 ions; maximum injection time was 100 ms. Up to 5 data-dependent MS2 spectra were acquired at 60,000 resolution. AGC target for MS2 was set to 1,000,000 ions. In order to reach this target, long MS2 injection times were allowed (500 ms). Unassigned or singly-charged ions were rejected and the dynamic exclusion option was enabled (duration: 20 s).

For the quantification of very low abundance ADP-ribosylated peptides, a similar method with a smaller scan range (400-900 m/z) was also used.

#### ETD (Electron Transfer Dissociation) Acquisitions

For localization of ADPr sites pure ETD and mixed HCD/ETD datasets were acquired on an Orbitrap Fusion instrument (Thermo Scientific). Our method for targeted acquisition of ETD spectra of modified precursors used the product ion trigger feature in the decision tree of a TopSpeed acquisition method. As they eluted, multiply-charged precursors were rapidly fragmented in HCD mode (low injection time: 30 ms; resolution 15,000; AGC target: 50,000) to screen a maximum number of precursors for diagnostic Adenine ions (typically among the strongest fragment ion signals, 136.062 Da). Upon detection of an intense diagnostic peak, the respective precursor was immediately isolated again and subjected to ETD (Rosenthal et al., 2015). Since the entire cycle time was held to a maximum of 3 s, the entire process from MS1, through screening, to ETD fragmentation took place well within the width of a chromatographic peak. The maximum MS2 injection time and AGC for ETD were set to 1000 ms and 500,000 ions in order to achieve single amino-acid resolution for particularly difficult precursors. Resolution was set to 30,000.

#### Data Analysis

Raw files were analyzed with MaxQuant proteomics suite of algorithms (version 1.5.3.17) (Cox and Mann, 2008), integrated with the search engine Andromeda (Cox et al., 2011).

For the analysis of localization of ADPr, the data were searched against the human proteome database (downloaded 09.10.2015 from UniProt) with the following parameters. The maximum allowed mass deviation was set to 4.5 ppm for precursor ions and 20 ppm for fragment ions; the minimum peptide length was set to 6 amino acids and the maximum number of missed cleavages was set to 5 with the maximum charge state 7. Variable modifications included oxidation (M), acetylation (Protein N-term and K), Amidation (C-term), ADP-ribosylation (DEKRSTCYNQHM) or phosphoribosylation (DEKRSTCYNQHM). The variable modification ADP-ribosylation allowed for neutral losses of adenine (m/z 136.0618); adenosine with loss of water (m/z 250.0935); AMP (m/z 348.0704); ADP (m/z 428.0367) and ADP-ribose (m/z 542.0684) (Hengel and Goodlett, 2012), with AMP listed first. FTMS top peaks per 100 Da were set to 20. For confident identification of ADP-ribosylation sites, we considered only ETD MS/MS spectra and required a minimum Andromeda score of 100, mass deviation smaller than 3 ppm after MaxQuant recalibration and a localization score above 0.9. In addition, we manually validated all the representative spectra by requiring extensive coverage of the peptide backbone fragment ions. For localization we required the clear presence of multiple high-intensity fragment ions pinpointing the modification site. Unlike the HCD spectra, ETD spectra do not contain ADP-ribose specific diagnostic ions, which are not generated in the ETD reaction, and are thus not available as a criterion for validation of spectra. In all triggered spectra, however, the adenine peak was observed in the HCD fragmentation of the same precursor.

#### Analysis of SILAC Experiments

For SILAC experiments on histones (Figure 1), HCD data were collected and searched against a human histone database (generated from a human proteome database downloaded on 09.10.2015 from UniProt) with the parameters given above with the following changes: Multiplicity was set to 2, with *Lys8* as the Heavy Label. Maximum labeled AAs were set to 7. Maximum missed cleavages were set to 6, and maximum charge was 7. The minimum peptide length was set to 6 amino acids. Variable modifications included acetylation (Protein N-term and K), methylation (KR) and ADP-ribosylation (S), since the localization of these ADPr sites was already well established with the preceding ETD experiments and the HCD data contained relatively little localization information.

For SILAC experiments of PARP-1 immunoprecipitation (Figure S2), HCD and triggered ETD data were collected and searched against the human proteome database (downloaded 09.10.2015 from UniProt) with the parameters given above for histones SILAC experiments with the following changes: Variable modifications included acetylation (Protein N-term) and ADP-ribosylation (DEKRSTCYNQHM).

For Figures 1B and 1G representative MS spectra were manually selected and annotated. For Figures S1A, S1G, and S2H evidence tables from MaxQuant were analyzed using Perseus software (http://www.perseus-framework.org) together with an in-house script to create the scatterplots comparing SILAC ratios versus total peptide intensities (left panels) and light versus heavy ADP-ribosylated peptide intensities (right panels). For Figures S1A and S1G, histones SILAC ratios and histones peptide intensities were plotted. For Figure S2H, PARP-1 SILAC ratios and PARP-1 peptide intensities were plotted.

#### Reanalysis of Published High-Quality Proteomics Datasets

For the reanalysis of published high-quality proteomics datasets, public raw files were searched against the human proteome database (downloaded 09.10.2015 from UniProt) with the parameters given above with the following changes: the maximum number of missed cleavages was set to 3 with the maximum charge state 7.

For the high-quality phosphoproteomics study of human stem cells (Phanstiel et al., 2011), variable modifications included acetylation (Protein N-term and K), and ADP-ribosylation (DEKRSTCYNQHM) and, since isobaric labeling was used, we selected the option “4plex iTRAQ” under the “Reporter Ion MS2” menu.

For the ADPr proteomics study based on enrichment of modified peptides with a macrodomain ADPr-binding module (Martello et al., 2016), variable modifications included acetylation (Protein N-term), Oxidation (M) and ADP-ribosylation (DEKRSTCYNQHM). We employed the MS/MS spectra generated by MaxQuant as the basis for our manual validation of spectra. To consider a peptide as modified on serine, we required the presence of fragment ions with either the intact ADP-ribose or phosphoribose (resulting from the loss of AMP) pointing to ADPr on serine. For localization we disregarded fragment ions for which it is impossible to distinguish between an original lack of modification and complete loss of ADPr during fragmentation. Manually validated spectra from the reanalysis of Martello et al., 2016 dataset are in Mendeley Data and are available at http://dx.doi.org/10.17632/pmvv5mdmrm.1.

Significantly enriched Gene Ontology terms of the serine ADP-ribosylated proteins identified after manual validation were determined using the PANTHER (protein annotation through evolutionary relationship) classification system (http://www.pantherdb.org; Mi et al., 2013) with the following parameters: Analysis type - PANTHER Overrepresentation Test (release 20160715), Annotation Version and Release Date - GO Ontology database Released 2016-10-27, Reference List - *Homo sapiens* (all genes in database), Annotation Dataset - GO biological process complete.

#### Localization-free Searching

We have previously observed prominent neutral loss behavior for ADP-ribosylated peptides undergoing HCD fragmentation (Sharifi et al., 2013). Among the losses observed, that of AMP and of the complete modifier have appeared most prominently. While the loss of AMP provides information about possible modifier localization, modeling it requires definition of the possible amino acids modified.

Modeling ADPr as an entirely labile variable modifier on the peptide C terminus has advantages as well. For one, we need make no assumptions about the amino acid specificity of ADPr – any peptide may be modified, regardless of composition. Second, assigning the modification to the terminus of the peptide means each possible peptide in the search space generates only two hypothetical spectra for matching, one with and one without the modifier, rather than a combinatorial set in which every permissible amino acid leads to a different hypothesis. Thus the search space is dramatically reduced and the search runs faster.

Since the HCD data provided by Martello et al. (2016) was collected with higher than typical sensitivity it is still more likely to contain the native series ions arising from complete loss of the modifier.

Searches were performed using the fast search engine Morpheus 1.68 (Wenger and Coon, 2013) – a version implementing neutral loss modeling, generously provided by Craig Wenger. The three HeLa HCD files provided (20140515_QE6_UPLC5_SCL_SA_Hela_PAR_PD_Rep1, 20140515_QE6_UPLC5_SCL_SA_Hela_PAR_PD_Rep2, 20140515_QE6_UPLC5_SCL_SA_Hela_PAR_PD_Rep3) were searched versus the same database of human proteins mentioned above with the following parameters: Tryptic peptides (no proline rule) - Miscleavages 3 – variable mods carbamidomethylation of C, oxidation of M, acetylation of protein N terminus, ADPRcompleteNL of C-term. Both precursor and fragment mass tolerances were set at 10 ppm and the precursor isotopic assignment was allowed to be off by up to 3.

These searches identified 1818 unique ADPr modified peptides and 16982 unique peptides without ADPr. The amino acid composition of these two sets of peptides is compared in Table S4.

### Data Availability

Mass spectrometry data have been deposited in the ProteomeXchange Consortium (http://proteomecentral.proteomexchange.org) via the PRIDE partner repository (Vizcaíno et al., 2014) with the dataset identifier ProteomeXchange: PXD005627.

The unprocessed image files used to prepare the figures in this manuscript and the manually validated spectra from the reanalysis of Martello et al., 2016 dataset are in Mendeley Data and are available at http://dx.doi.org/10.17632/pmvv5mdmrm.1.

## Author Contributions

I.M., J.J.B., and I. Ahel conceived the study. J.J.B. designed and performed the SILAC experiments and acquired and analyzed mass spectrometric data. J.J.B., P.F., E.B., R.Z., and Q.Z. performed protein purification, mutagenesis, and in vitro assays. I.G.-S. made reagents and performed supporting studies. T.C. and I. Atanassov performed bioinformatic analyses with the help of I.M. and J.J.B. Gene ontology (GO) analysis was performed by E.B. I.M. wrote the manuscript with contributions from all the authors.

## Supplemental Information

Document S1. Figures S1–S4

Table S1. MaxQuant Evidence Tables from SILAC Experiments Performed in This Work, Related to Figures 1, S1, and S2 Evidence_PARP1ko_Hx: all identified peptide features from SILAC experiments from Figure 1A. ADPr_evidence_PARP1ko_Hx: ADPr identified peptide features from SILAC experiments from Figure 1A. Evidence_HPF1ko_Hx: all identified peptide features from SILAC experiments from Figure 1F. ADPr_Evidence_HPF1ko_Hx: ADPr identified peptide features from SILAC experiments from Figure 1F. Evidence_HPF1ko_PARP1: all identified peptide features from SILAC experiments from Figure S2G. ADPr_Evidence_HPF1ko_PARP1: ADPr identified peptide features from SILAC experiments from Figure S2G.

Table S2. Identified Serine ADPr Sites after In Vitro ADP-Ribosylation Reactions in the Presence of HPF1, Related to Figures 1 and 2 Analysis of serine ADP-ribosylation on different substrates. In vitro ADP-ribosylation assays were performed with NAD+, DNA and recombinant PARP-1 or PARP-2 obtained from *E. coli* in the presence of recombinant HPF1 WT. Serine ADPr sites that were also identified in vivo are indicated. ^a^Serine ADPr sites were detected by using high resolution ETD Mass Spectrometry. ^b^Previously reported in Leidecker et al, Nat Chem Biol 2016. ^c^Reported in this work.

Table S3. Identification of Serine ADPr Sites after Re-analysis of the Dataset Generated by Martello et al. (2016), Related to Figure 4 List of serine ADPr sites after manual inspection of spectra pinpointing ADPr on serine residues. To consider a peptide as modified on serine, we required the presence of fragment ions with either the intact ADP-ribose or phosphoribose (resulting from the loss of AMP) pointing to ADPr on serine. For localization we disregarded fragment ions for which it is impossible to distinguish between an original lack of modification and complete loss of ADPr during fragmentation. See Experimental procedures for further details. Representative high-resolution HCD fragmentation MS/MS spectra of localized serine ADP-ribosylation sites are available at http://dx.doi.org/10.17632/pmvv5mdmrm.1

Table S4. Occurrence of the Different Amino Acids at N Termini of ADPr Peptides Relative to Non-ADPr Peptides, Obtained by Reprocessing the Dataset Generated by Martello et al. (2016), Related to Figure 4 Analysis: Analysis of the occurrence of the different amino acids at the Nterminus of the ADPr peptides and nonADPr peptides. ADPr (FDR 1%): List of identified 1818 unique ADPr modified peptides after localization-free searching of the dataset generated by Martello et al. nonADPr (FDR 1%): List of identified 16982 unique peptides without ADPr after localization-free searching of the dataset generated by Martello et al. All searches of this table were performed using the fast search engine Morpheus 1.68. See Experimental procedures for further details.

Document S2. Article plus Supplemental Information
