# Supplementary material for: Serine ADP-Ribosylation Depends on HPF1
Source: Mol Cell. 2017 Mar 2;65(5):932–940.e6. doi: 10.1016/j.molcel.2017.01.003 (PMC5344681; doi:10.1016/j.molcel.2017.01.003)
Supplement: Document S1. Figures S1–S4 [file mmc1.pdf]

Molecular Cell, Volume *65*

Supplemental Information

Serine ADP-Ribosylation Depends on HPF1

Juan José Bonfiglio, Pietro Fontana, Qi Zhang, Thomas Colby, Ian Gibbs-Seymour, Ilian Atanassov, Edward Bartlett, Roko Zaja, Ivan Ahel, and Ivan Matic

Bonfiglio et al., Figure S1

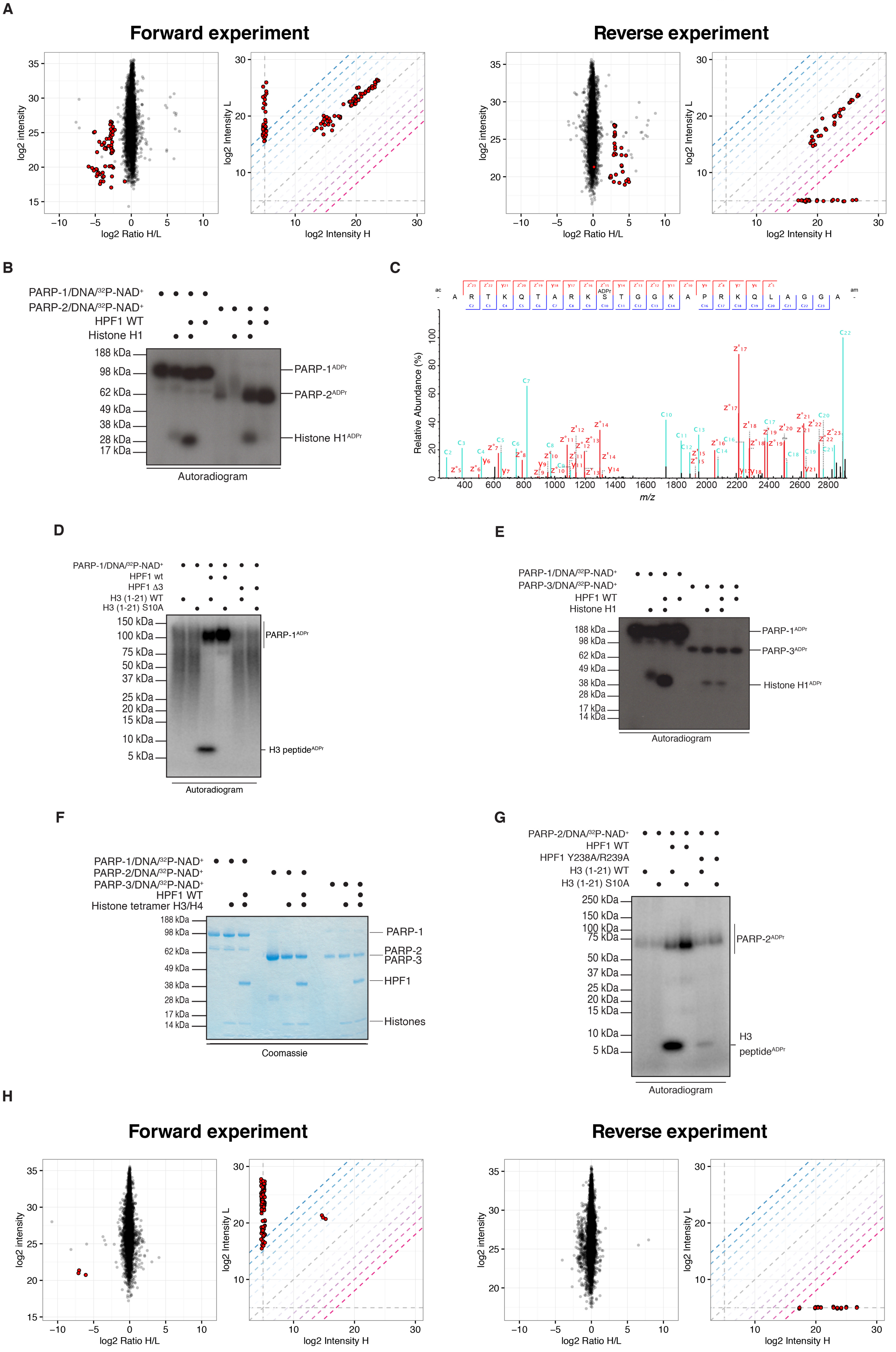

Figure S1 (Related to Figure 1).

Histone serine ADPr is dependent on HPF1.

A. Data analysis of SILAC experiments (both Forward and Reverse) comparing WT and Δ*PARP-1* U2OS cells upon 10 minutes of 2 mM H_2O_2-induced DNA damage. Log_2 of summed peptide intensities were plotted against log_2 Heavy/Light SILAC ratios for forward and reverse experiments respectively (left hand panels) as were Log_2 of light ADPr peptide intensities versus log_2 heavy ADPr peptide intensities (right hand panels). ADPr peptides are marked in red.

B. Analysis of recombinant H1 ADP-ribosylation by PARP-1 or PARP-2 in the presence of HPF1. In vitro ADP-ribosylation assays were performed with $^{32}P\text{-}NAD^+$, DNA and recombinant PARP-1 or PARP-2 obtained from *E.coli* in the presence or absence of recombinant HPF1 and/or recombinant H1. Samples were resolved by SDS-PAGE and analyzed by autoradiography.

C. High-resolution ETD fragmentation spectrum of a synthetic H3 peptide (H3 [1-21] WT) subjected to an in vitro ADP-ribosylation assay in the presence of NAD^+, DNA, PARP-1 and HPF1. H3 peptide is modified by ADP-ribose on Serine 10.

D. Analysis of the ADP-ribosylation of two different variants of synthetic peptides corresponding to the amino acids 1-21 of human H3. In vitro ADP-ribosylation assays were performed with $^{32}P\text{-}NAD^+$, DNA and recombinant PARP-1 obtained from *E.coli* in the presence or absence of recombinant HPF1 WT or a Δ3HPF1 mutant. Samples were resolved by SDS-PAGE and analyzed by autoradiography.

E. Analysis of recombinant H1 ADP-ribosylation by PARP-1 or PARP-3 in the presence of HPF1. In vitro ADP-ribosylation assays were performed with ^{32}P-NAD^{+}, DNA and recombinant PARP-1 or PARP-3 obtained from *E.coli* in the presence or absence of recombinant HPF1 and/or recombinant H1. Samples were resolved by SDS-PAGE and analyzed by autoradiography.

F. Coomassie stain of SDS-PAGE gel from which autoradiogram of Figure 1E was obtained.

G. Analysis of the ADP-ribosylation of two different variants of synthetic peptides corresponding to the amino acids 1-21 of human H3. In vitro ADP-ribosylation assays were performed with ^{32}P-NAD^{+}, DNA and recombinant PARP-2 obtained from *E.coli* in the presence or absence of recombinant HPF1 WT or a Y238A/R239A mutant. Samples were resolved by SDS-PAGE and analyzed by autoradiography.

H. Data analysis of SILAC experiments (both Forward and Reverse) comparing WT and Δ*HPF1* U2OS cells upon 10 minutes of 2 mM H_2O_2-induced DNA damage. Log_2 of summed peptide intensities were plotted against log_2 Heavy/Light SILAC ratios (left hand panels) for forward and reverse experiments, as were log_2 of light ADPr peptide intensities against log_2 heavy ADPr peptide intensities (right hand panels). ADPr peptides are in red.

Bonfiglio et al., Figure S2

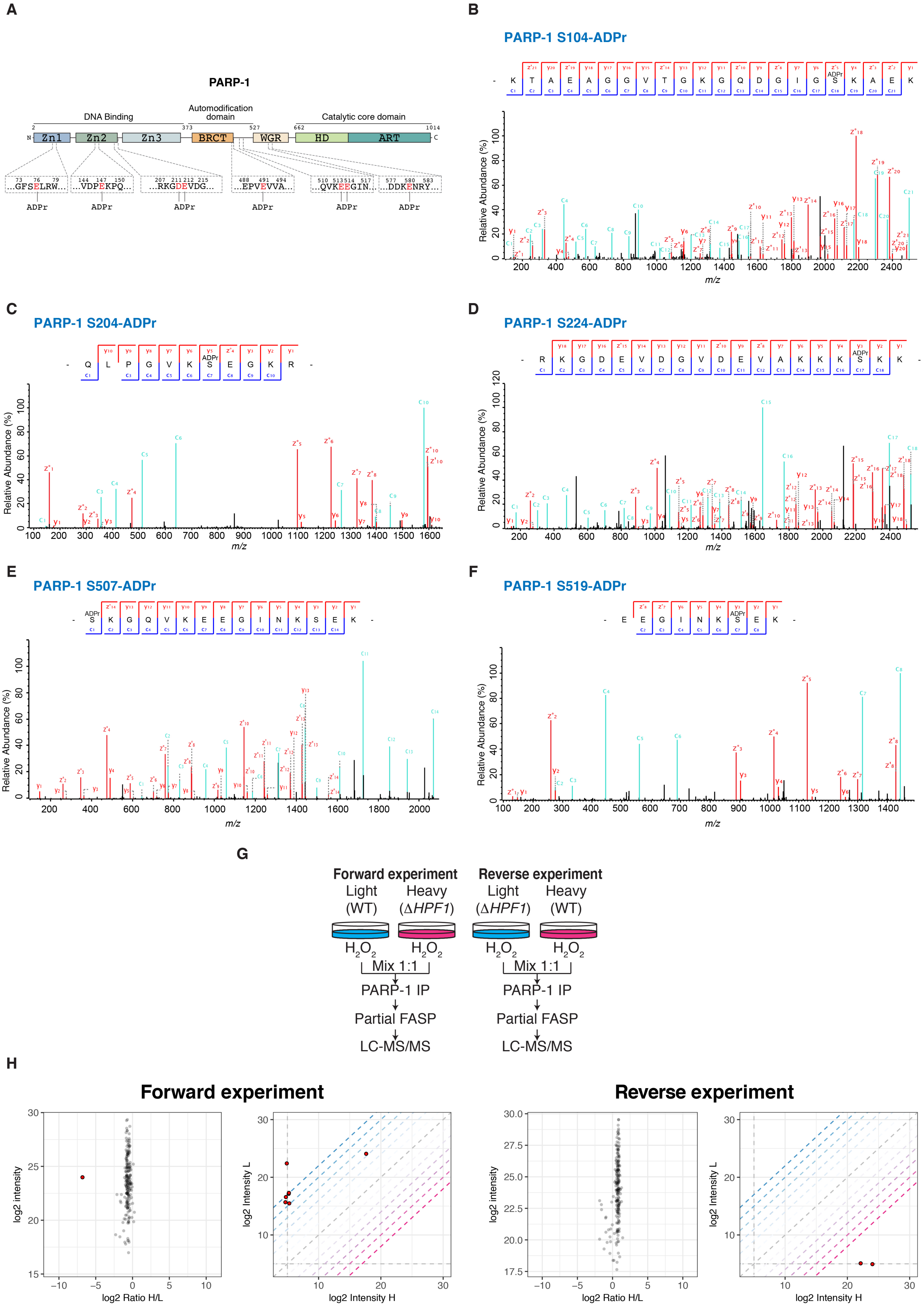

Figure S2 (Related to Figure 2).

HPF1 changes PARP-1 amino acid specificity towards serine.

A. Schematic representation of PARP-1. Identified PARP-1 automodification on acidic residues are depicted. Samples were treated with a Nudix hydrolase. Zn I, II and III: zinc-finger domains. BRCT: breast cancer suppressor protein-1 domain. WGR: WGR domain (named after a well-conserved region of the amino acid sequence, Trp-Gly-Arg). HD: alpha-helical subdomain. ART: ADP-ribosyl transferase subdomain

B. High-resolution ETD fragmentation spectrum of a PARP-1 peptide modified by ADP-ribose on serine 104.

C. High-resolution ETD fragmentation spectrum of a PARP-1 peptide modified by ADP-ribose on serine 204.

D. High-resolution ETD fragmentation spectrum of a PARP-1 peptide modified by ADP-ribose on serine 224.

E. High-resolution ETD fragmentation spectrum of a PARP-1 peptide modified by ADP-ribose on serine 507.

F. High-resolution ETD fragmentation spectrum of a PARP-1 peptide modified by ADP-ribose on serine 519.

G. Schematic representation of the SILAC-based strategy to quantify PARP-1 ADPr marks upon 10 minutes of 2 mM H_2O_2-induced DNA damage in WT and Δ*HPF1* U2OS cells.

H. Data analysis of SILAC experiments (both Forward and Reverse) comparing WT and Δ*HPF1* U2OS cells upon 10 minutes of 2 mM H_2O_2-induced DNA damage. Log_2 of summed PARP-1 peptide

intensities were plotted against $\log_2$ Heavy/Light SILAC ratios of PARP-1 peptides (left panels). Log_2 of light PARP-1 ADPr peptide intensities were plotted against $\log_2$ heavy ADPr PARP-1 peptide intensities (right panels). ADPr peptides are in red.

Bonfiglio et al., Figure S3

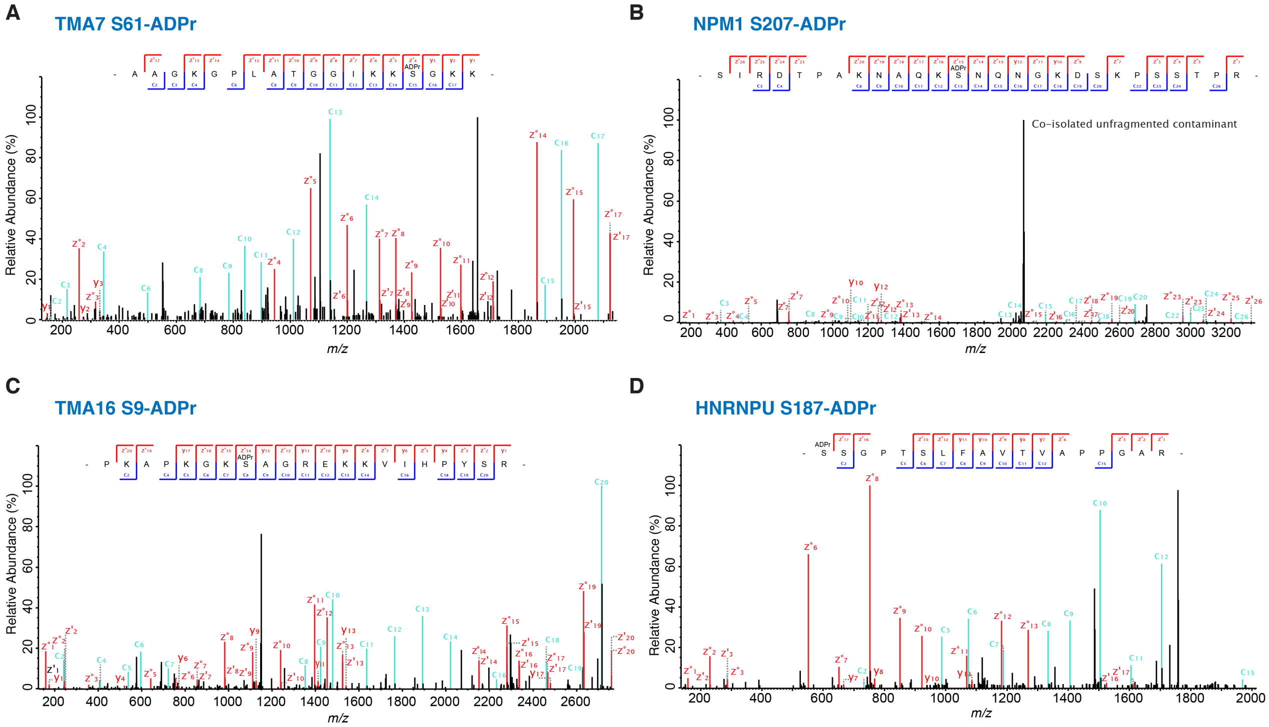

Figure S3 (Related to Figure 3).

Additional targets of serine ADPr identified from fractions depleted of histones.

A. High-resolution ETD fragmentation spectrum of a TMA7 peptide modified by ADP-ribose on serine 61.

B. High-resolution ETD fragmentation spectrum of a NPM1 peptide modified by ADP-ribose on serine 207. The intense peak at 2071.9447 m/z corresponds to the +1 charge state of a co-isolated contaminant (m/z 691.3891 +3) that did not fragment due to its low initial charge state.

C. High-resolution ETD fragmentation spectrum of a TMA16 peptide modified by ADP-ribose on serine 9.

D. High-resolution ETD fragmentation spectrum of a HNRNPU peptide modified by ADP-ribose on serine 187.

Bonfiglio et al., Figure S4

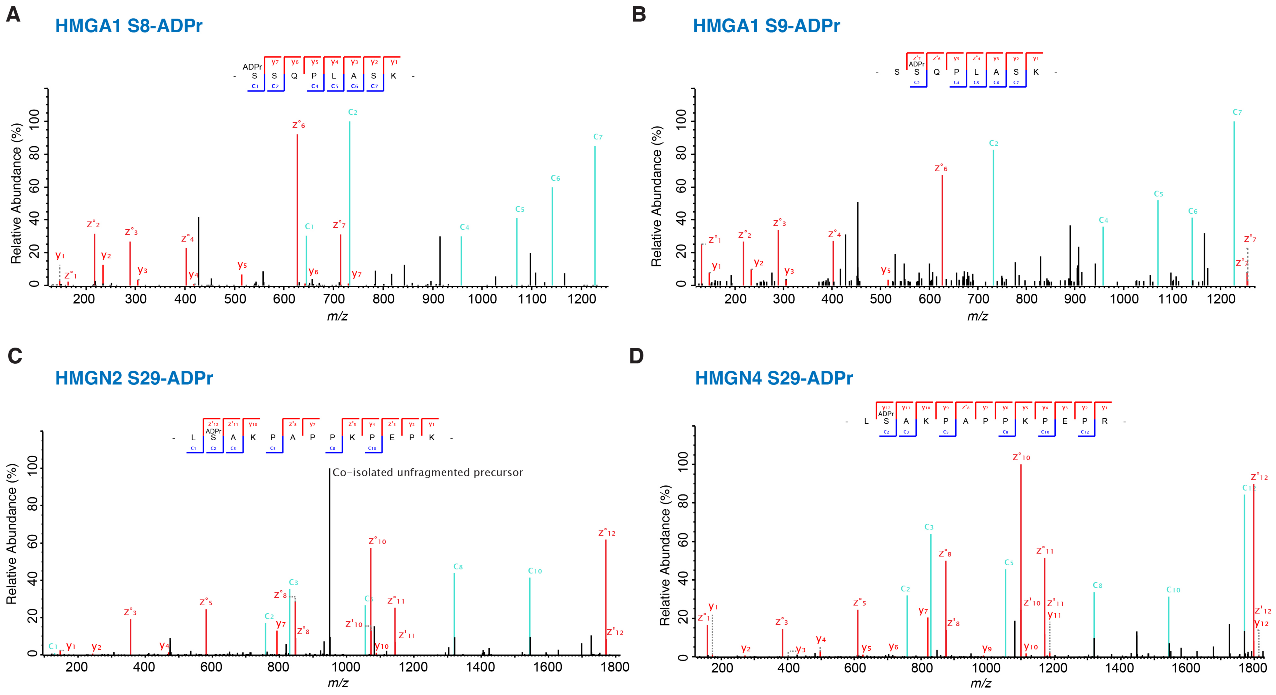

Figure S4 (Related to Figure 4).

In vivo serine ADPr sites of High Mobility proteins can be reproduced in vitro in the presence of PARP-1 and HPF1

A. High-resolution ETD fragmentation spectrum of a HMGA1 peptide modified by ADP-ribose on serine 8.

B. High-resolution ETD fragmentation spectrum of a HMGA1 peptide modified by ADP-ribose on serine 9.

C. High-resolution ETD fragmentation spectrum of a HMGN2 peptide modified by ADP-ribose on serine 29. The intense peak at 949.4824 m/z corresponds to the charge-reduced state of a co-isolated precursor (m/z 475.2276 2+) that did not fragment due to its lower charge.

D. High-resolution ETD fragmentation spectrum of a HMGN4 peptide modified by ADP-ribose on serine 29.

Table S1 (Related to Figures 1, S1 and S2).

MaxQuant evidence tables from SILAC experiments performed in this work.

- *Evidence_PARP1ko_Hx*: all identified peptide features from SILAC experiments from **Figure 1A**.
- *ADPr_evidence_PARP1ko_Hx*: ADPr identified peptide features from SILAC experiments from **Figure 1A**.
- *Evidence_HPF1ko_Hx*: all identified peptide features from SILAC experiments from **Figure 1F**.
- *ADPr_Evidence_HPF1ko_Hx*: ADPr identified peptide features from SILAC experiments from **Figure 1F**.
- *Evidence_HPF1ko_PARP1*: all identified peptide features from SILAC experiments from **Figure S2G**.
- *ADPr_Evidence_HPF1ko_PARP1*: ADPr identified peptide features from SILAC experiments from **Figure S2G**.

Table S2 (Related to Figures 1 and 2).

Identified serine ADPr sites after in vitro ADP-ribosylation reactions in the presence of HPF1.

Analysis of serine ADP-ribosylation on different substrates. In vitro ADP-ribosylation assays were performed with NAD^+, DNA and recombinant PARP-1 or PARP-2 obtained from *E.coli* in the presence of recombinant HPF1 WT. Serine ADPr sites that were also identified in vivo are indicated. [a]Serine ADPr sites were detected by using high resolution ETD Mass Spectrometry [b]Previously reported in Leidecker et al, Nat Chem Biol 2016. [c]Reported in this work.

Table S3 (Related to Figure 4).

Identification of serine ADPr sites after re-analysis of the dataset generated by Martello et al.

List of serine ADPr sites after manual inspection of spectra pinpointing ADPr on serine residues. To consider a peptide as modified on serine, we required the presence of fragment ions with either the intact ADP-ribose or phosphoribose (resulting from the loss of AMP) pointing to ADPr on serine. For localization we disregarded fragment ions for which it is impossible to distinguish between an original lack of modification and complete loss of ADPr during fragmentation. See Experimental procedures for further details.

Representative high-resolution HCD fragmentation MS/MS spectra of localized serine ADP-ribosylation sites are available at http://dx.doi.org/10.17632/pmvv5mdmrm.1

Table S4 (Related to Figure 4).

Occurrence of the different amino acids at N-termini of ADPr peptides relative to non-ADPr peptides obtained by reprocessing the dataset generated by Martello et al.

- *Analysis*: Analysis of the occurrence of the different amino acids at the N-terminus of the ADPr peptides and nonADPr peptides.
- *ADPr (FDR 1%)*: List of identified 1818 unique ADPr modified peptides after localization-free searching of the dataset generated by Martello et al.
- *nonADPr (FDR 1%)*: List of identified 16982 unique peptides without ADPr after localization-free searching of the dataset generated by Martello et al. All searches of this table were performed using the fast search engine Morpheus 1.68. See Experimental procedures for further details.
